# 

**Published:** 1909-01

**Authors:** 


### CONTENTS.

ORIGINAL COMMUNICATIONS—

Annual Address of the President of Fulton County Medical Society. By A. W. Stirling, M. D., C. M., Edin; D. P. H., Eng; Atyanta, Ga. .......... 511

The Results of Vaccine Therapy in Acute and Chronic Infections. By J. Edgar Paullin, B. A., M. D., Atlanta, Ga. .......... 516

Complications of Gonorrhoea in Women. By F. G. Hodgson, M. D., Atlanta, Ga. .......... 525

Tuberculosis. By George Brown, M. D., Atlanta, Ga. .......... 538

Adenoids. By Dr. Ridley, M. D., Atlanta, Ga. .......... 533

Hunger and Thirst and Some More of That Sort. By Dr. C. A. F. Lindmorme, Atlanta, Ga... 537

EDITORIALS .......... 541

NEWS AND NOTES .......... 549

BOOK REVIEWS .......... 551

